# The Youth Anxiety Measure for DSM-5 (YAM-5): An Updated Systematic Review of its Psychometric Properties

**DOI:** 10.1007/s10578-024-01712-3

**Published:** 2024-06-05

**Authors:** Iván Fernández-Martínez, Peter Muris

**Affiliations:** 1 Department of Health Psychology, Centro de Investigación de la Infancia/Child Research Center, Miguel Hernández University, Av. de la Universidad S/N, 03202 Elche, Alicante Spain; 2 Maastricht University, Maastricht, The Netherlands; 3 Stellenbosch University, Stellenbosch, South Africa

**Keywords:** Youth anxiety measure for DSM-5 (YAM-5), Anxiety disorders, Phobias, Children and adolescents, Psychometric properties, Review

## Abstract

The Youth Anxiety Measure for DSM-5 (YAM-5) is a self- and parent-report scale specifically developed to assess symptoms of major anxiety disorders (part 1 or YAM-5-I) and specific phobias/agoraphobia (part 2 or YAM-5-II) in children and adolescents in terms of the contemporary psychiatric classification system. Since its introduction, the measure has been increasingly used in research, making it feasible to provide a summary of its psychometric properties. The present article presents a systematic review of 20 studies that employed the YAM-5, involving 5325 young participants. Overall, the results supported the hypothesized factor structure of both parts of the measure, although there were also some studies that could not fully replicate the original five-factor model of YAM-5-I. The internal consistency of the YAM-5 was generally high for the total scores of both parts, while reliability coefficients for the subscales were more variable across studies. Research also obtained evidence for other psychometric properties, such as test–retest reliability, parent–child agreement, convergent/divergent validity, and discriminant validity. Results further revealed that girls tend to show significantly higher anxiety levels on the YAM-5 than boys. Overall, these findings indicate that the YAM-5 is a promising tool for assessing symptoms of anxiety disorders including specific phobias in young people. Some directions for future research with the YAM-5 and recommendations regarding the use of the measure are given.

## Introduction

Anxiety is one of the most common psychological problems in children and adolescents, with the prevalence of anxiety disorders being estimated between 5 and 20% [1, 2]. Untreated anxiety can lead to persistent trajectories over time and the development of other mental health conditions (e.g., mood disorders) as well as impaired functioning in various life domains [3]. Therefore, proper assessment and early identification is key, as this can facilitate providing adequate interventions. The usefulness of rating scales as an efficient method for assessing mental health problems in children and adolescents has been underscored, along with the importance of relying on instruments with adequate and empirically validated psychometric properties [4, 5].

Several rating scales have been developed that assess symptomatology related to anxiety disorders and phobias in young people, and these have been widely used in research as well as clinical practice [6]. Some of them measure more generic and global aspects of anxiety and fear, such as the Revised Children's Manifest Anxiety Scale (RCMAS [7]), the State-Trait Anxiety Inventory for Children (STAIC [8]), and the Fear Survey Schedule for Children-Revised (FSSC-R [9]), while other scales more specifically address various types fear and anxiety symptoms as described by current psychiatric nosology systems such as the Diagnostic and Statistical Manual of Mental Disorders (DSM).

Examples of the latter category are the Spence Children's Anxiety Scale (SCAS [10] or its modified version, the Revised Child Anxiety and Depression Scale [11], which besides anxiety also measures depressive symptoms) and the Screen for Child Anxiety Related Emotional Disorders (SCARED [12]), which both have been demonstrated to be reliable and valid indices of anxiety symptoms in children and adolescents [e.g., 13, 14]. However, the SCAS and the SCARED were developed more than 25 years ago and since then a number of modifications have been made in the DSM [15], which are not covered by these scales. To begin with, the SCAS includes a subscale measuring symptoms of obsessive–compulsive disorder, which is no longer classified as an anxiety disorder in the latest edition of DSM, whereas the SCARED incorporates school phobia, which is not considered as a separate anxiety category in this psychiatric classification system. Furthermore, both the SCAS and the SCARED do not incorporate selective mutism, which the DSM has added to the category of anxiety disorders, given compelling evidence showing that anxiety plays a key role in this condition [16]. A final limitation of these scales pertains to the deficient measurement of fears and phobias: the SCARED does not assess this type of anxiety at all, while the SCAS only measures a limited number of specific fears (by means of its *physical injury fears* subscale). Meanwhile, agoraphobia is increasingly regarded as a separate diagnostic entity which is no longer exclusively linked to panic disorder [17], while other specific fears and phobias are quite heterogeneous but can be grouped in overarching categories [18] that are quite prevalent in children and adolescents [19] and often indicative for the presence of other internalizing problems [20].

In order to obtain a rating scale that assesses symptoms of the full range of anxiety disorders and phobias in young people in terms of the contemporary classification system, Muris et al. [21] developed the Youth Anxiety Measure for DSM-5 (YAM-5). The development of the measure was based on a meticulous review of the DSM-5 criteria and an item construction procedure involving international panels of clinicians and researchers with extensive expertise in childhood anxiety. The YAM-5 covers a number of anxiety disorders that are also measured with the SCAS and SCARED (i.e., social anxiety disorder, separation anxiety disorder, generalized anxiety disorder, and panic disorder), but includes new subscales covering symptoms of selective mutism, agoraphobia, and the most common types of specific phobias (while excluding obsessive–compulsive disorder). It is important to note that although the DSM-5 was recently subjected to a revision (DSM-5-TR [22]), no further changes were made with regard to the classification of anxiety disorders, implying that the YAM-5 is still up to date.

The YAM-5 is a self- and parent-report scale to assess anxiety and fear symptoms in young people from age 8 onwards. It consists of two parts, each comprising five subscales/factors. The first part (YAM-5-I) contains 28 items that cover symptoms of five major anxiety disorders: separation anxiety disorder (6 items; e.g., “I get frightened if my parents leave the house without me”), social anxiety disorder (6 items; e.g., “I find it scary to eat or drink if other people are looking at me”), generalized anxiety disorder (6 items; e.g., “I worry about a lot of things”), selective mutism (4 items; e.g., “At school I don’t speak to the teacher at all”), and panic disorder (6 items; e.g., “I suffer from anxiety or panic attacks”). The second part (YAM-5-II) comprises 22 items measuring symptoms of agoraphobia/situational phobias (6 items; e.g., “I am afraid of being in crowded places with lots of people”), animal phobias (5 items; e.g., “I am afraid of dogs”), blood-injection-injury phobias (3 items; e.g., “I am afraid of getting an injection”), natural environment phobias (4 items; e.g., “I am afraid of the dark”), and other phobias (4 items; e.g., “I am afraid that I will feel sick and have to vomit”). All items of the YAM-5 are rated on a four-point Likert scale (0 = never, 1 = sometimes, 2 = often, and 3 = always). Ratings are summed across relevant items to obtain total and subscale scores. Total scores for parts I and II range from 0 to 84 and from 0 to 66, respectively, with higher scores reflecting higher levels of anxiety disorder and phobia symptoms.

A first psychometric evaluation of the YAM-5 in non-clinical and clinically referred children and adolescents from The Netherlands [21] revealed that the scale showed good face validity (which means that clinicians were able to link its items to DSM-defined anxiety disorders), sufficient to good internal consistency, reasonable concurrent and discriminant validity, and acceptable parent–child agreement. Furthermore, this initial study indicated that the measure was easily completed by the children/adolescents and their parents, hence supporting its suitability as an index for quantifying young people’s anxiety symptoms in a cost-effective manner.

Altogether, the YAM-5 aims to improve the accuracy of screening and assessment of fear and anxiety problems in children and adolescents according to the current DSM-5 nosology, and to facilitate the application of a multi-informant approach to assess this type of internalizing psychopathology in both clinical and research settings. Since its development, researchers have used the YAM-5 to assess fear and anxiety symptoms in children and adolescents for various purposes. In some studies, the psychometric properties of the measure were examined (e.g., [23]), whereas other investigations employed the scale as an index of fear/anxiety to explore its correlation with other psychological constructs (e.g., [24]) or used it as an outcome measure in a treatment or intervention trial (e.g., [25]). Some years ago, Çankaya and Cevik [26] provided a preliminary test review of the YAM-5 concluding that the measure seems to display good reliability and validity, while also noting that further research on the scale is needed to firmly establish its psychometric qualities. Since then more studies have been published that used the YAM-5 and so the time has come to provide an update of this research and review the main findings. This could help to gain more insight in the usability and usefulness of the YAM-5 in clinical and research settings as well as highlight directions for future studies aimed at improving the (psychometrics of the) measure.

Thus, the main goal of the current study was to conduct a systematic review of the reliability and validity of the YAM-5, considering both parts of the measure (YAM-5-I and YAM-5-II) as well as the self- and parent-report versions. Specifically, this study aimed to synthesize the results of the extant literature, focusing on various psychometric aspects such as (a) the factor structure; (b) the internal consistency; (c) test–retest reliability; (d) validity (i.e., convergent, divergent, and discriminant validity); and (e) other properties of interest such as parent–child agreement and age and sex differences.

## Method

### Literature Search and Selection of Studies

**Fig. 1:**
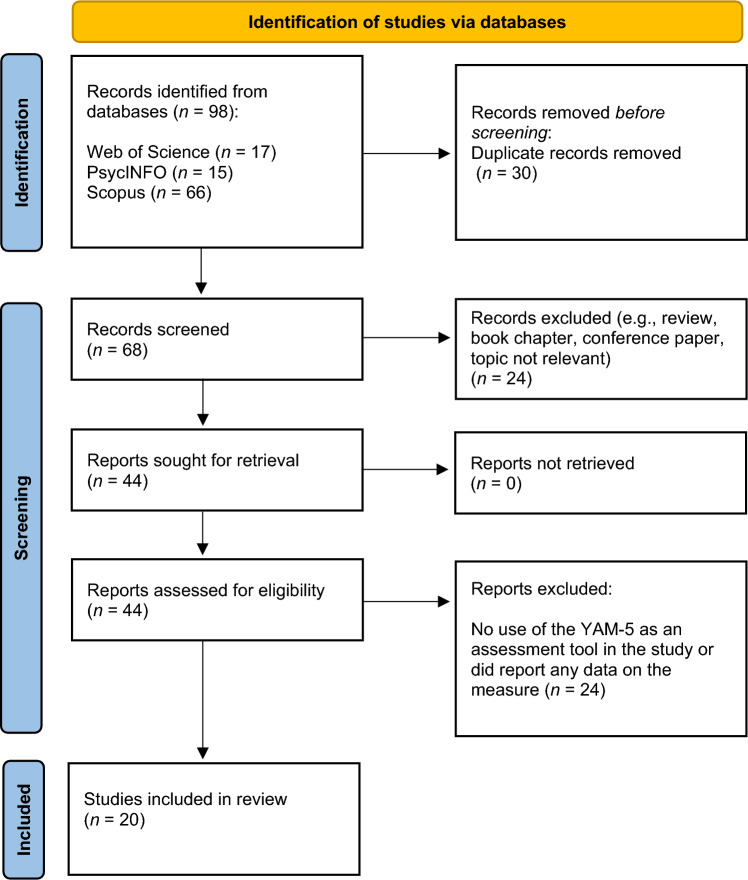
PRISMA 2020 flow diagram of the review process

A literature review was conducted in order to find articles that reported on empirical studies using (parts of) the YAM-5. The search was carried out in November 2023 in databases such as Web of Science, Scopus, and PsycINFO, with "Youth Anxiety Measure for DSM-5" or "YAM-5" in the title, abstract, or text as the key search terms. Inclusion criteria were: (a) articles describing an original study that was published in an English, peer-reviewed journals; and (b) use of the YAM-5 as an assessment instrument, thereby providing some data about the measure (e.g., mean scores, factor structure, reliability coefficients, or correlations with other constructs). Articles for which the in-text was not available in English or that had not been published in scientific journals (e.g., thesis dissertations, conference papers, book chapters) or that did not report original data of YAM-5 (e.g., review studies) were excluded. After applying the inclusion/exclusion criteria, eventually a total of 20 articles were retained. No overlapping samples were identified. It is important to note that of all these studies, only four (i.e., [21, 23, 27, 28]) were included in the previously mentioned test review of the YAM-5 as conducted by Çankaya and Cevik [26]. Figure 1 shows the Preferred Reporting Items for Systematic Reviews and Meta-Analysis (PRISMA) flow diagram [29] of the review process followed in the current study.

## Results

### Overview of the Studies

Table 1 shows an overview of the 20 studies included in the review. A total of 5325 participants were involved across the studies, coming from a variety of countries including The Netherlands, Iran, Portugal, Spain, Algeria, and the United States. The samples generally comprised a proportionate number of boys and girls, covering an age range between 8 and 19 years. Of the 20 studies, most were conducted in non-clinical populations, except for four studies that also included young clinically referred participants [e.g., 21, 25, 30, 31].

**Table 1:** Description of the studies included in the current systematic review of the YAM-5

References	Type	Sample size (boys/girls)	Country (language of the measure)	Age range	Mean age (SD)	Type of population	Version	YAM-5 Part
Fuentes-Rodriguez et al. [30]	Psychometric	48 (19/29)	Spain (Spanish)	14–17	15.29 (.96)	Clinical	Self-report	I (only Social anxiety disorder subscale)
Garcia-Lopez et al. [27]	Psychometric	505 (238/267)	Spain (Spanish)	13–17	14.94 (1.25)	Non-clinical	Self-report	I
Gillig and Bighas [32]	Intervention	238 (several gender identities)	United States (English)	12–18	15.0 (1.72)	Non-clinical	Self-report	I (only Generalized anxiety disorder subscale)
Gillig et al. [33]	Intervention	41 (several gender identities)	United States (English)	12–19	15.5 (2.03)	Non-clinical	Self-report	I (only Generalized anxiety disorder subscale)
Gillig et al. [34]	Intervention	181 (several gender identities)	United States (English)	12–18	15.1 (2.03)	Non-clinical	Self-report	I (only Generalized anxiety disorder subscale)
Ivaki et al. [35]	Psychometric	Sample 1: 520 (262/258)	Iran (Persian)	9–14	*Boys : 11.52 (1.71); Girls: 11.16 (1.87)	Non-clinical	Self-report	I
		Sample 2: 557 (277/280)	Iran (Persian)	9–14	*Boys: 11.38 (1.74); Girls: 11.42 (1.78)	Non-clinical	Self-report	I
Maleki et al. [25]	Intervention	31 (19/12)	Iran (Persian)	NR	Experimental group: 13.06 (0.96) Control group: 13.50 (1.09)	Clinical	Self-report	I, II (only total scores)
Martinez-Snyder et al. [37]	Psychometric	331 (only reported percentages; 71% girls)	United States (English)	NR	17.11 (0.79)	Non-clinical	Self-report	I
Mohajerin et al. [31]	Intervention	93 (42/51)	Iran (Persian)	12–17	*Group 1 (*n* = 46): 14.86 (1.21) Group 2 (*n* = 47): 14.86 (1.21)	Clinical	Self-report	I, II (only full scale score that combined both parts)
Muris et al. [21]	Psychometric	Sample 1: 132 (56/76)	The Netherlands (Dutch)	12–17	14.8 (1.09)	Non-clinical	Self-report	I, II
		Sample 2: 64 (24/40)	The Netherlands (Dutch)	8–18	12.4 (3.1)	Clinical	Self-report and parent-report	I, II
Muris et al. [24]	Correlational	87 (26/61)	The Netherlands (Dutch)	12–18	15.28 (0.51)	Non-clinical	Self-report	I (only total score)
Muris et al. [28]	Correlational	187 (84/103)	The Netherlands (Dutch)	8–12	10.5 (1.0)	Non-clinical	Self-report	I, II
Muris et al. [40]	Correlational	118 (53/65)	The Netherlands (Dutch)	12–15	13.31 (0.67)	Non-clinical	Self-report	I, II (only total scores)
Muris et al. [47]	Correlational	Sample 1: 106 (54/52)	The Netherlands (Dutch)	12–17	13.65 (1.24)	Non-clinical	Self-report	I (only total score)
		Sample 2: 52 (15/33; 4NR)	The Netherlands (Dutch)	11–16	13.31(1.29)	Non-clinical	Self-report	I (only total score)
Muris et al. [48]	Correlational	157 (50/107)	The Netherlands (Dutch)	12–18	15.33 (1.31)	Non-clinical	Self-report	I (only total score)
Oliveira et al. [36]	Psychometric	300 (112/188)	Portugal (Portuguese)	11–16	13.13 (1.29)	Non-clinical	Self-report	I
		Pilot study: 45 (19/26)	Portugal (Portuguese)	12–15	13.73 (1.27)	Non-clinical	Self-report	I
Simon et al. [23]	Psychometric	414 (217/197)	The Netherlands (Dutch)	8–12	10.49 (1.04)	Non-clinical	Self-report	I, II
Simon et al. [39]	Psychometric	678 (329/349)	Algeria (Arabic)	8–12	10.74 (1.04)	Non-clinical	Self-report	I, II
Simon et al. [41]	Correlational	63 (22/41)	The Netherlands (Dutch)	8–13	10.38 (1.57)	Non-clinical	Self-report	I, II (only full scale score that combined both parts)
Soltani et al. [38]	Psychometric	377 (174/185)	Iran (Persian)	12–18	15.10 (1.35)	Non-clinical	Self-report	I, II

*YAM-5* Youth Anxiety Measure for DSM-5, *NR* Not Reported, *SD* Standard Deviation

*Mean age (and standard deviation) for the total sample was not reported

In terms of study design, there were nine studies that aimed to examine the psychometric properties of the measure, six correlational studies that employed the YAM-5 as an index of anxiety within the context of some other psychological construct, and five were intervention studies examining the impact of an intervention on different problems, including anxiety symptoms as assessed with the YAM-5. All studies relied on the self-report version of the YAM-5: 11 of these investigations only used part I, while other studies administered both parts of the measure. Only one study [21] also obtained data on the parent version of the measure. Four studies relied on a single subscale of the measure of the YAM-5-I, such as the social anxiety or generalized anxiety subscale [30, 32–34], nine studies made use of various subscales scores of only the YAM-5-I [27, 35–37] or both parts [21, 23, 28, 38, 39], while seven studies only employed the full scale score of the YAM-5 (Table 1).

The YAM-5 was originally developed in Dutch and during the initial validation procedure (which involved international experts) also translated into English following a translation–back-translation procedure. In the past years, the measure has also become available in other languages including Spanish [27], Persian [35], Portuguese [36], and Arabic [39], but to our knowledge, none of translations has resulted in the removal or substantial changes of its items for cultural or other reasons.

### Psychometric Properties of the YAM-5 Self-Report Version

#### Factor Structure

Since the publication of the original study [21], seven studies have been conducted that examined the factor structure of the self-report version of the YAM-5 by means of exploratory factor analysis (EFA) and/or confirmatory factor analysis (CFA). As can be seen in Table 2, all studies were carried out in non-clinical populations and included part I of the YAM-5 (i.e., major anxiety disorders), while three of these studies also focused on part II (i.e., specific phobias and agoraphobia).

**Table 2:** Main findings of studies examining the factor structure of the YAM-5

References	Country	Type of population (age range)	Version (YAM-5 part)	Method	Number of factors found (part I/II)	Main findings compared to the original factor structure [21]
Garcia-Lopez et al. [27]	Spain	Non-clinical (13–17)	Self-report (I)	EFA/CFA	6	Removal of 11 items; support for 4 of the original factors, while separation anxiety symptoms were split in two different factors
Ivaki et al. [35]	Iran	Non-clinical (9–14)	Self-report (I)	EFA/CFA	5	Support for the original factor structure, after removing one item
Martinez-Snyder et al. [37]	United States	Non-clinical (NR)	Self-report (I)	SEM	4	Support for the original factor structure, but only after discarding the selective mutism subscale. A slightly better fit for a three-factor model was found, after also removing the panic disorder subscale
Oliveira et al. [36]	Portugal	Non-clinical (11–16)	Self-report (I)	EFA/CFA	4	Removal of five items; support for most of the original factors, but one factor consisted of a mix of social anxiety and generalized anxiety items
Simon et al. [23]	The Netherlands	Non-clinical (8–12)	Self-report (I, II)	CFA	5/5	Support for the original factor structure of both parts. For the YAM-5-I, the CFA showed a better fit after removing one item
Simon et al. [39]	Algeria	Non-clinical (8–12)	Self-report (I, II)	CFA	5/3	Original factor structure failed to converge for both parts. After moving agoraphobia to Part I and combining it with panic disorder, hypothesized model was largely confirmed for YAM-5-I. For YAM-5-II, three factors were found: animal phobias, natural environment phobias, and blood-injection-injury and other phobias combined
Soltani et al. [38]	Iran	Non-clinical (12–18)	Self-report (I, II)	CFA	5/5	Support for the original factor structure of both parts

*YAM-5* Youth Anxiety Measure for DSM-5, *EFA* Exploratory Factor Analysis, *CFA* Confirmatory Factor Analysis, *SEM* Structural Equation Modeling, *NR* Not Reported

##### Studies Supporting the Original Factor Structure of the YAM-5

Three of the seven studies found support for the hypothesized factor structure of the YAM-5. Two studies, which were conducted in Dutch [23] and Iranian [38] populations, obtained evidence for the existence of the original five factors in both the YAM-5-I and the YAM-5-II using CFA; while one other investigation by Ivaki et al. [35], which also relied on an Iranian sample, found support for the original five-factor model of YAM-5-I using EFA/CFA. The study by Simon et al. [23] suggested a somewhat better fit for the YAM-5-I when removing one item from the panic disorder subscale (i.e., item 17: “When I panic, I am afraid that I could die”), whereas Ivaki et al. [35] noted an improved fit when removing one item from the selective mutism subscale (i.e., item 2: “At school I don't speak to the teacher at all”).

##### Variations in Factor Structure

Meanwhile, it should be noted that other studies conducted with the YAM-5-I in Spanish [27] and Portuguese [36] samples have obtained a slightly different factor structure. For example, the study by Garcia-Lopez et al. [27] used EFA/CFA to obtain a 6-factor solution, but only after removing a considerable number of 11 items. The factors were generally in keeping with the original YAM-5 factors, but separation anxiety disorder appeared to split into two separate factors, apparently related to ‘fear of being alone’ and ‘fear of abandonment’. Oliveira et al. [36] conducted EFA to obtain a 4-factor solution after removing five items. Again, most factors were as hypothesized but the social anxiety and generalized anxiety disorder items appeared to merge on one factor. This 4-factor model of the YAM-5-I was confirmed by means of CFA and showed a better fit in this Portuguese sample than the original five-factor model.

Martinez-Snyder et al. [37], who conducted their study in United States, focused on Part I of the YAM-5. After removing the selective mutism subscale, factorial validity was found for the remaining subscales (i.e., separation anxiety, social anxiety, panic, and generalized anxiety subscales) using Structural Equation Modeling. However, a slightly better fit was even noted for a three-factor model after also disregarding the panic disorder subscale.

A final study by Simon et al. [39] employed CFA to investigate the factor structure of the YAM-5 in a large sample of Algerian school children. The results indicated that the hypothesized factor structure failed to converge for both parts of the measure. Interestingly, by moving the agoraphobia subscale from part II to part I, this problem was largely repaired. For part I, the CFA revealed a good fit for a five-factor model that included a combined panic disorder–agoraphobia subscale as one of the factors, while for part II, a satisfactory three-factor structure emerged which was composed of animal phobias, natural environment phobias, and blood-injection-injury + other phobias.

#### Internal Consistency

Nineteen of the included studies reported internal consistency coefficients for the measure, most frequently Cronbach's alpha (Table 3). Results in both non-clinical and clinical populations generally indicate reliability coefficients ranging from .85 to .93 for the full YAM-5 scale, which combines both parts of the measure [23, 31, 40, 41].

**Table 3:** Internal consistency reliability of the self-report version of the YAM-5 as reported in various studies

Study	Internal consistency coefficients*
Fuentes-Rodriguez et al. [30]	YAM-5-I: SOC: 88^a^
Garcia-Lopez et al. [27]	YAM-5-I: total score: .84^a^, subscales: SEP1: .81, SEP2: .58, SM: .58^a^, SOC: .79^a^, PAN: .84^a^, GAD: .85^a^
Gillig & Bighas [32]	YAM-5-I: GAD: .90
Gillig et al. [33]	YAM-5-I: GAD: .84
Gillig et al. [34]	YAM-5-I: GAD: .92
Ivaki et al. [35]– Sample 1	YAM-5-I: total score: .90^a^, SEP: .77, SM: .71^a^, SOC: .77, PAN: .84, GAD: .82
Ivaki et al. [35]– Sample 2	YAM-5-I: total score: .87^a^, SEP: .77, SM: .70^a^, SOC: .73, PAN: .77, GAD: .76
Martinez-Snyder et al. [37]	YAM-5-I: SEP: .79, SOC: .82, PAN: .90, GAD: .87
Mohajerin et al. [31]	YAM-5 full scale**: .85
Muris et al. [21]– Sample 1	YAM-5-I: total score: .93, SEP: .80, SM: .65, SOC: .81, PAN: .76, GAD: .85 YAM-5-II: total score: .86, ANI: .66, NATENV: .47, BII: .65, SITAGO: .74, OTH: .47
Muris et al. [21]–Sample 2	YAM-5-I: total score: .92, SEP: .84, SM: .55, SOC: .81, PAN: .82, GAD: .83 YAM-5-II: total score: .86, ANI: .59., NATENV: .61, BII: .62, SITAGO: .67, OTH: .41
Muris et al. [24]	YAM-5-I: total score: .93
Muris et al. [28]	YAM-5-I: total score: .92, SEP: .84, SM: .41, SOC: .76, PAN: .77, GAD: .78 YAM-5-II: total score: .89, ANI: .67., NATENV: .61, BII: .68, SITAGO: .72, OTH: .72
Muris et al. [40]	YAM-5 full scale**: .90 YAM-5-I: total score: .88 YAM-5-II: total score: .80
Muris et al. [47]–Sample 1	YAM-5-I: total score: .93
Muris et al. [47]–Sample 2	YAM-5-I: total score: .89
Muris et al. [48]	YAM-5-I: total score: .92
Oliveira et al. [36]	YAM-5-I: total score: .88^a^, SEP: .77, SM: .68, PAN: .86^a^, SOC + GAD: .88
Oliveira et al. [36]–Pilot sample	YAM-5-I: total score: .87, SM: .62, PAN: .81
Simon et al. [23]–Time 1	YAM-5 full scale**: .92 YAM-5-I: total score: .91, SEP: .75, SM: .50, SOC: .77, PAN: .79, GAD: .82 YAM-5-II: total score: .84, ANI: .63, NATENV: .59, BII: .70, SITAGO: .59, OTH: .55
Simon et al. [23]–Time 2	YAM-5 full scale**: .93 YAM-5-I: total score: .92, SEP: .78, SM: .61, SOC: .81, PAN: .83, GAD: .86 YAM-5-II: total score: .87, ANI: .67, NATENV: .61, BII: .77, SITAGO: .61, OTH: .58
Simon et al. [39]	YAM-5-I: total score: .85, SEP: .64, SM: .42, SOC: .55, PAN: .65, GAD: .64 YAM-5-II: total score: .86, ANI: .69, NATENV: .59, BII: .47, SITAGO: .67, OTH: .53
Simon et al. [41]	YAM-5 full scale**: .88
Soltani et al. [38]	YAM-5-I: total score: .84, SEP: .61, SM: .55, SOC: .59, PAN:.66, GAD: .70 YAM-5-II: total score: .78, ANI: .53, NATENV: .47, BII: .51, SITAGO: .57, OTH: .38

*YAM-5* Youth Anxiety Measure for DSM-5, *SEP* separation anxiety disorder, *SM* selective mutism, *SOC* social anxiety disorder, *PAN* panic disorder, *GAD* generalized anxiety disorder, *ANIM* animal phobia, *NATENV* natural environment phobia, *BII* blood–injection–injury phobia, *SITAGO* situational phobia/agoraphobia, *OTH* other phobias. *SOC* + *GAD* factor containing a mix of 9 SOC and GAD items

*All studies reported Cronbach’s alpha coefficient, except for the studies by Oliveira et al. [36] and Simon et al. [23], which computed McDonalds's Omega

**Combining items of Part I and Part II

^a^Coefficients obtained after discarding some items from the original (sub)scale

With regard to Part I (major anxiety disorders) of the self-report YAM-5, reliability for the total score ranged from .84 to .93, while coefficients for the subscales varied considerably across studies, ranging between .41 (selective mutism subscale) and .92 (generalized anxiety disorder). In general, the selective mutism subscale exhibited fairly low reliability coefficients across studies (mostly < .60), although there were also some exceptions [21, 23, 35, 36].

Part II (specific phobias and agoraphobia) of the self-report YAM-5 has been less frequently investigated. The reliability values for the total score of this part varied between .78 and .89, while coefficients of the subscales were generally lower than those obtained for Part I, ranging from .38 (other phobias subscale) to .77 (blood-injection-injury type subscale). In particular, the other phobias subscale demonstrated low reliability in most studies (.38–.58) [21, 23, 38, 39].

Two final remarks can be made regarding the internal consistency of the YAM-5. First, the reliability data obtained in studies that relied on clinical populations [21, 30, 31] were largely similar to those reported in non-clinical samples. Second, no systematic differences in reliability were noted across various countries. Considerably lower internal consistency coefficients have been reported in studies conducted in Iran [38] and Algeria [39], which might indicate that the YAM-5 is less applicable to children and adolescents in some non-Western countries [but see e.g., 35].

#### Test–Retest Reliability

In this review, three studies were found that reported evidence on the test–retest reliability of the YAM-5 in community samples (Table 4). The study by Soltani et al. [38], which relied on a small sample of young people from Iran (*n* = 30), evaluated the 4-weeks test–retest reliability of the measure using Spearman correlations. The results showed test–retest correlations ranging from .82 (panic disorder subscale) to .96 (YAM-5-I total score, animal phobia, blood–injection–injury phobia). A further study by Simon et al. [23], which was conducted in a Dutch sample (*n* = 181), also investigated the test–retest reliability of the YAM-5 for a period of four weeks, and noted significant positive Pearson correlations, ranging from .54 (selective mutism) to .90 (YAM-5 full scale score). Finally, in another investigation of the same research group [41], a significant positive Pearson correlation of .79 for the YAM-5 full scale score was reported in a small sample of school children (*n* = 63), after a 30-day period.

**Table 4:** Test–retest reliability of the YAM-5

Study	Test–retest correlations*
Simon et al. [23]^a^	YAM-5 full scale**: .90 YAM-5-I: total score: .86, SEP: .75, SM: .54, SOC: .81, PAN: .81, GAD: .78 YAM-5-II: total score: .89, ANI: .85, NATENV: .85, BII: .83, SITAGO: .81, OTH: .73
Simon et al. [41]^a^	YAM-5 full scale**: .79
Soltani et al. [38]^b^	YAM-5-I: total score: .96; SEP: .90; SM: .88; SOC: .93; PAN: .82, GAD: .92 YAM-5 II: total score: .93; ANI: .96; NATENV: .95; BII: .96; SITAGO: .88; OTH: .93

*YAM-5* Youth Anxiety Measure for DSM-5, *SEP* separation anxiety disorder, *SM* selective mutism, *SOC* social anxiety disorder, *PAN* panic disorder, *GAD* generalized anxiety disorder, *ANIM* animal phobia, *NATENV* natural environment phobia, *BII* blood–injection–injury phobia, *SITAGO* situational phobia/agoraphobia, *OTH* other phobias

*All studies provided test–retest reliability of the YAM-5 self-report version for a four-week/30-day period

**Combining items of Part I and Part II

^a^Studies using Pearson's correlations

^b^Study using Spearman correlations

#### Convergent and Divergent Validity

The convergent and divergent validity of the YAM-5 self-report version have been examined in nine studies included in this review (Table 5). Four studies analyzed both parts of the YAM-5, while five studies only focused on Part I. Non-clinical samples predominated, with only two studies including clinically referred youth [21, 30].

**Table 5:** Summary of studies examining the validity of the YAM-5

Study	Variables and measures	Evidence of convergent/divergent validity?	Evidence of discriminant validity?
Fuentes-Rodriguez et al. [30]	Social Anxiety: SAS-A and SPAI-B Depression: CDI	Yes	Yes (by comparing socially anxious with non-socially anxious adolescents)
Garcia-Lopez et al. [27]	Social Anxiety: SAS-A and SPAI-B	Yes	NI
Ivaki et al. [35]	Anxiety: RCMAS Depression: CDI	Yes	NI
Martinez-Snyder et al. [37]	Anxiety: SCAS, SCARED Depression: SMFQ Experiential avoidance: AFQ-Y Intolerance of uncertainty: IUSC	Yes	NI
Muris et al. [21]	Anxiety disorders diagnoses: Junior SCID Internalizing and externalizing problems: CBCL and YSR	Yes	Yes (by comparing clinically referred youths with anxiety disorders with non-clinical and clinically referred youths with other problems)
Muris et al. [28]	Anxiety: SCAS, trait anxiety version of the STAIC Fear/fearfulness: Short form of the FSSC-R Depression: CDI Selective Mutism: SMQ	Yes	Yes (by comparing high versus low anxiety groups based on the SCAS)
Oliveira et al. [36]	Fear of Failure and Criticism: FSSC-R Internalizing problems: YSR Interpersonal Sensitivity and Anxiety: BSI	Yes	NI
Simon et al. [23]	Anxiety: SCARED Behavioral Inhibition: BIQ	Yes	Yes (by comparing high versus low anxiety groups based on the SCARED)
Soltani et al. [38]	Internalizing and externalizing problems: CBCL	Yes	NI

*YAM-5* Youth Anxiety Measure for DSM-5, *NI* Not Investigated, *SCAS* Spence Children's Anxiety Scale, *SMQ* Selective Mutism Questionnaire, *STAIC* State-Trait Anxiety Inventory for Children, *FSSC-R* Fear Survey Schedule for Children-Revised, *CDI* Children’s Depression Inventory, *CBCL* Child Behavior Checklist, *SCARED* Screen for Child Anxiety Related Emotional Disorders, *BIQ-C-SF* Behavioral Inhibition Questionnaire for Children–Short Form, *Junior SCID* Junior version of the Structured Clinical Interview for DSM-5, *YSR* Youth Self-Report, *SAS-A* Social Anxiety Scale for Adolescents, *SPAI-B* Brief version of the Social Phobia and Anxiety Inventory, *RCMAS* Revised Children’s Manifest Anxiety Scale, *BSI* Brief Symptom Inventory, *SMFQ* Short Mood and Feelings Questionnaire, *AFQ-Y* Avoidance and Fusion Questionnaire for Youth, *IUSC* Intolerance of Uncertainty Scale for Children

In general, studies tend to provide strong support for the convergent/divergent validity of both parts of the measure, showing a meaningful pattern of correlations with a wide range of constructs and instruments (Table 5). Notably, significant positive correlations were obtained with other measures of anxiety and fear (e.g., SCAS, SCARED, RCMAS, and FSSC-R), as well as with scales assessing related constructs such as depression (e.g., Child Depression Inventory–CDI [42]) or internalizing symptoms (e.g., Child Behavior Checklist–CBCL or Youth Self-Report–YSR [43]; Brief Symptom Inventory–BSI [44]). In contrast, correlations were weaker or even non-significant with measures of less related constructs (e.g., externalizing problems as measured by the CBCL or YSR).

For example, the study by Muris et al. [28] demonstrated that YAM-5-I major anxiety disorders were more strongly associated with specific anxiety measures (e.g., STAIC, SCAS), while YAM-5-II phobias were more strongly linked to an index of fear/fearfulness (e.g., FSSC-R). Positive associations were also found between the YAM-5 and an index of depression (i.e., CDI), and this was especially true for the YAM-5-I total score and the subscales measuring generalized and social anxiety. The strong connection between YAM-5-I, depression (CDI), and other specific anxiety measures (e.g., RCMAS, SCARED) was also supported by other studies [23, 35, 37]. Furthermore, other studies found additional evidence for the convergent and/or divergent validity of the YAM-5 through relations with measures of internalizing or externalizing symptoms [21, 36, 38].

It is worth noting that the selective mutism subscale, which was incorporated in the YAM-5-I following its inclusion as a new anxiety disorder in the DSM-5 [15, 16], generally showed only modest to moderate relationships with other measures of anxiety or internalizing problems (e.g., [23, 27, 36]). However, it was also noted that this subscale was strongly and positively correlated with another index of selective mutism (Selective Mutism Questionnaire [45], see [28]).

Finally, the study by Muris et al. [21] found evidence of convergent validity in a sample of clinically referred children and adolescents, reporting significant positive correlations between the total scores of both parts of the YAM-5 with young people’s anxiety symptoms as assessed with the Junior SCID [46], a clinician-administered interview for DSM-classifications.

#### Discriminant Validity

As seen in Table 5, four studies provided evidence for the discriminant validity of the YAM-5, with two studies only involving non-clinical participants and two studies also including clinically referred youth. In general, support has been found for the discriminant validity of the YAM-5: the scale was demonstrated useful to correctly identify children with high or even clinically significant anxiety levels. For instance, the study by Muris et al. [28] showed that non-clinical children who scored in the clinical range on the SCAS had higher levels of symptoms of major anxiety disorders in YAM-5-I and phobias in YAM-5-II, compared to children who scored in the normative range. Similarly, Simon et al. [23] observed that children classified as clinically anxious based on the SCARED had significantly higher scores on the YAM-5 full scale score as well as on the total scores of YAM-5-I and YAM-5-II. Furthermore, the study by Muris et al. [21] revealed that clinically referred youths with anxiety disorders scored higher on the YAM-5-I (major anxiety disorders) scale as compared to young people in two control groups (i.e., clinically referred youths with other problems and non-clinical controls). Finally, the study by Fuentes-Rodriguez et al. [30], which included both clinical and non-clinical participants and only focused on the social anxiety subscale of YAM-5-I, revealed support for the high accuracy of this subscale as a screening measure to identify adolescents at risk for social anxiety disorder by means of ROC curve analysis.

#### Other Findings of Interest

Several studies examined relationships between anxiety as measured by the YAM-5 and other psychological constructs (e.g., self-compassion, guilt, shame, child avoidance, depression, neuroticism). These studies generally found significant and theoretically meaningful correlations between anxiety symptoms as measured by the YAM-5 and these psychological concepts in children and adolescents [e.g., 24, 40, 41, 47, 48].

Sex differences in YAM-5 scores were investigated in eight of the included studies. With regard to sex differences, the majority of studies using the YAM-5 reported significantly higher levels of anxiety and phobia symptoms in girls than in boys, not only on the total score(s) of the measure [23, 24, 27, 28, 40, 41] but also on most of its subscales [28]. However, there are also studies that did not observe such sex differences in YAM-5 scores [30, 35], whereas one investigation by Ivaki et al. [35] even showed a reversed pattern with boys scoring significantly higher on a subscale (i.e., selective mutism) as compared to girls.

Studies generally failed to find significant correlations between age and YAM-5 scores, suggesting that this demographic variable has little impact on the anxiety and fear scores as obtained with the measure. One exception was the study by Muris et al. [28] which reported small but significant negative correlations between age and separation anxiety and panic symptoms (YAM-5-I) and other phobias (YAM-5-II), suggesting that the frequency of these symptoms slightly decreased with increasing age.

Finally, measurement invariance was examined in one study [27], which focused on the YAM-5-I administered in adolescents aged 13–17 years. The results demonstrated good measurement invariance across age and gender.

### Psychometric Properties of the YAM-5 Parent-Report Version

#### Internal Consistency

The internal consistency reliability of the parent version of the YAM-5 was only evaluated in the study by Muris et al. [21], who examined a sample of clinically referred children and adolescents and their parents (a total of 63 parents completed the parent-version of the YAM-5 of whom 74% were mothers). Cronbach’s alpha coefficients for the total score were found to be .91 for Part I and .77 for Part II. For the subscales of the YAM-5-I, the internal consistency coefficients ranged from .84 (separation anxiety) to .87 (generalized anxiety), with the exception of the selective mutism subscale, which showed an alpha of .64. For the subscales of the YAM-5-II, most internal consistency coefficients were fairly low (i.e., between .35 and .53), except for blood–injection–injury phobia for which a Cronbach’s alpha of .86 was reported.

#### Validity

Muris et al. [21] also examined the validity of the YAM-5 parent-report version using the CBCL and the Junior SCID interview instruments. The authors found support for the convergent and divergent validity of the measure, showing positive significant Pearson correlations between the parent-version of the YAM-5-I (major anxiety disorders) and YAM-5-II (phobias) and internalizing problems as measured by the CBCL, while noting no significant correlations between these YAM-5 scores and externalizing problems. In addition, significant positive correlations between the parent-version YAM-5-I and YAM-5-II total scores and young people’s anxiety symptoms as reported during the Junior SCID interview.

#### Child-Parent Agreement

Two studies evaluated the parent–child agreement of the YAM-5 [21, 38], demonstrating that the correspondence between these two informants was satisfactory. The study by Soltani et al. [38], which relied on a subsample of a larger non-clinical Iranian population (*n* = 47), reported Spearman correlation coefficients of 0.68 for the YAM-5-I total score and 0.85 for the YAM-5-II total score. Furthermore, Muris et al. [21] provided support for the parent–child agreement in the clinical sample, reporting positive and significant Pearson correlations of 0.69 and 0.70 for the YAM-5-I and YAM-5-II total scores, respectively. Additionally, correlations ranged between 0.42 (selective mutism) and 0.73 (separation anxiety) for various subscales, with most correlations being around 0.60 or higher.

## Discussion

The main objective of this research was to conduct a systematic review of the psychometric properties of the YAM-5, which is the only scale that assesses anxiety and fear symptoms as defined by the latest version of the DSM [15]. This review encompasses 20 studies, involving 5325 participants aged 8–19 years from various countries, including The Netherlands, Iran, Portugal, Spain, Algeria, and the United States. Findings showed that most studies were conducted in non-clinical populations, but there are also data from four studies incorporating clinically referred participants [21, 25, 30, 31]. All studies utilized the self-report version of the YAM-5, with the majority of studies only including part I of the measure. This indicates that since its release to the scientific community, the scale has generated considerable research interest. In the meantime, the number of studies using the scale could also be qualified as limited given the large number of studies that are published in the domain of childhood anxiety, and perhaps this is due to the fairly small changes in the DSM as a result of which most researchers stick with pre-existing DSM-IV-based measures (i.e., SCAS and SCARED).

The review highlights the need for further research on both parts of the measure, with a specific focus on the assessment of fear and anxiety symptoms in clinical populations and obtaining more information on the psychometric properties of the parent version. This study reveals that most researchers prefer to use part I of the YAM-5, which assesses symptoms related to major anxiety disorders. This may reflect that these anxiety problems are more frequently studied as compared to specific phobias (which are addressed in the second part of the measure). Meanwhile, research tends to indicate that specific fears (and phobias) are highly prevalent, have an early onset, and may predict the development of later anxiety and other internalizing disorders [49]. Therefore, YAM-5-II is also valuable, as it provides a comprehensive assessment of a wide range of specific fears.

Seven investigations have examined the factor structure of the YAM-5 self-report version, using exploratory factor analysis (EFA) and/or confirmatory factor analysis (CFA) in different cultural contexts. Overall, studies supported the existence of the anxiety disorder and phobia factors as proposed in the original study [21]. While some studies fully confirmed the original five-factor models of Part I and Part II, other investigations noted some variations with a slightly different number of factors and/or the removal of subscales and items. This indicates the need to further investigate the factor structure of the YAM-5 and explore cultural and methodological factors that may account for the observed variations, as has been suggested in previous studies with other DSM-based anxiety measures [e.g., SCAS, 14].

The studies that examined the internal consistency of the YAM-5 self-report version generally showed high reliability coefficients for the full scale score and the separate total scores of YAM5-I and YAM-5-II. The Cronbach’s alphas of the subscales of part I were mostly in order, but it was noticeable that the selective mutism subscale often displayed lower internal consistency. This could be explained by the low frequency of this (early childhood) anxiety problem in young people and the limited number of items that compose this subscale [21]. One way to solve this problem would be to create a separate module for selective mutism, which could be used by researchers who are specifically interested in this type of anxiety problem. The reliability coefficients of the YAM-5-II subscales (specific phobias and agoraphobia) showed considerable variability and were generally lower than those noted for the subscales in Part I. Although specific phobias can be grouped together in separate categories, the fears within each category remain by definition ‘specific’ and hence are less inter-related, making it logical that they produce lower reliability coefficients. This is particularly true for the other phobias subscale which can best be seen as a residual category.

Another aspect of reliability of the YAM-5 that certainly needs further examination is the test–retest reliability, which pertains to the degree to which scores on a measure remain unchanged when assessed on different time points. So far, three studies have evaluated this psychometric aspect of the YAM-5 in non-clinical samples. The results were quite favorable showing that the anxiety and fear scores as obtained with this scale were fairly stable over a four-week period [23, 38, 41].

The convergent and divergent validity of the total scale and subscales of the YAM-5 (of both the self-report and parent-report version) were also supported across various studies. The results showed that YAM-5 scores were correlated in a theoretically meaningful way with other scales assessing anxiety and fear (e.g., SCAS, RCMAS, SCARED, FSSC-R), internalizing and externalizing problems (e.g., CBCL, YSR), and depression (e.g., CDI). Additionally, a number of studies reported that YAM-5 scores were significantly related to a variety of psychological constructs (e.g., self-compassion, guilt, shame, child avoidance, depression, neuroticism [24, 40, 41, 47, 48]), confirming the association between childhood anxiety and these emotional, cognitive, behavioral, and personality variables. Support for the discriminant validity of the YAM-5 self-report version was also provided by four studies involving non-clinical as well as clinical samples [21, 23, 28, 30], indicating that the scale can be effectively used to identify children and adolescents exhibiting high or clinically significant levels of anxiety.

The cross-sectional data collected so far with both parts of the YAM-5 self-report version suggest that age has little or no impact on young people’s fear and anxiety levels. However, longitudinal studies examining trajectories of fear and anxiety in children and adolescents of course provide a better picture of the course of these symptoms throughout development [e.g., 50]. With regard to gender, most studies documented significantly higher fear and anxiety symptoms for girls than for boys, which is consistent with what has been reported in previous research [e.g., 51]. Furthermore, the results of the study by Garcia-Lopez et al. [27] demonstrated good measurement invariance for the YAM-5 self-report version across age and gender, which indicates that scores on the measure can be interpreted in the same way irrespective of these demographic variables.

Until now, few studies have evaluated the psychometric properties of the parent-report version of the YAM-5. Available evidence indicated that the internal consistency reliability of the YAM-5 (sub)scales completed by parents was comparable to that noted for the self-report version. Furthermore, the child-parent agreement was satisfactory, and there was also support for the concurrent validity of the parent version. Still, further research on the parent version is warranted given the importance of assessing young people’s mental health problems from the parents' perspective, apart from self-reports, even when discrepancies between informants are noticeable [4].

### Limitations and Future Lines of Research

The present findings should be interpreted with caution given a number of limitations of this review. First, there is a possibility that because of the strict search procedure, some original studies were not detected and included (e.g., due to specific inclusion/exclusion criteria, databases used). For instance, this review did not consider non-English articles published in scientific journals and grey literature (e.g., dissertations, conference papers), and this may have introduced a selection bias. Second, there was notable heterogeneity among studies in terms of populations, cultures, settings, and methods, which may account for variability in the results across studies. Furthermore, the main asset of the YAM-5 may also be its weakest point: the measure is strongly based on the DSM-5, a psychiatric classification system grounded in the Western society that has been criticized because of its lack of cross-cultural validity [52]. So far, the psychometric evaluation of the YAM-5 in non-Western countries has indicated that the scale may also be useful for assessing fear and anxiety symptoms of children and adolescents in other cultures, but further explorations of the transcultural similarities and diversities of data collected with the YAM-5 would be welcome.

With regard to direction for future research, more attention should be devoted to the psychometric evaluation of YAM-5-II (agoraphobia and specific phobias) and the parent version which have been less frequently studied. In addition, more studies are needed to further generate evidence by comparing clinical and non-clinical children and adolescents aged 8 to 18 years with diverse cultural and socioeconomic backgrounds. Other areas that are currently underexplored are the test–retest reliability, parent–child agreement, and measurement invariance. Moreover, the collection of more clinical data will be helpful to further explore the discriminant validity and treatment sensitivity of the measure, and to establish solid clinical cut-off scores, all of which would enhance its usability in clinical practice. In addition, the suggestion of moving the selective mutism subscale to a separate module needs empirical exploration: for example, one could investigate the factor structure of the YAM-5-I with and without the selective mutism subscale in future studies.

## Summary

In summary, the YAM-5 is a standardized rating scale for the assessing the frequency/intensity of several domains of anxiety symptoms in children and adolescents according to the current DSM-criteria. This easy-to-administer measure may be useful for screening purposes, facilitate classification in conjunction with the administration of a standardized diagnostic interview, or also make it possible to monitor changes in symptoms due to treatment and intervention. Furthermore, the YAM-5 allows for a multi-informant assessment approach (i.e., self- and parent-report) and flexible employment of various forms (see Appendix) to tailor the measurement to the researcher’s or clinician’s needs. For instance, instead of using the full scale, it is possible to administer Part I with/without the selective mutism subscale or use a somewhat extended version of Part I that adds the agoraphobia subscale of Part II. This review indicates that there is a steadily increasing amount of evidence to support the reliability and validity of the measure, but more research in clinical settings, of the parent version, and on specific psychometrics aspects (e.g., test–retest reliability, treatment sensitivity) is certainly needed.

## Data Availability

There are no other datasets associated with this systematic review beyond those presented in the manuscript and its appendix.

## Appendix

Child version of the YAM-5, scoring key, and possibilities for combining the subscales of the measure.

### Instructions and Items Included in the YAM-5-I and YAM-5-II

What are you to do? On the following pages there are statements that have to do with being afraid. Read every statement and fill in either never, sometimes, often or always as it applies to you. Don’t skip any question.

### YAM-5 Scoring Key

#### Yam-5-I

**Table:**

Separation anxiety disorder	1 + 6 + 10 + 15 + 19 + 24
Selective mutism	2 + 11 + 20 + 25
Social anxiety disorder	3 + 7 + 12 + 16 + 23 + 28
Panic disorder	4 + 8 + 13 + 17 + 21 + 26
Generalized anxiety disorder	5 + 9 + 14 + 18 + 22 + 27

#### Yam-5-II

**Table:**

Animal phobias	1 + 3 + 9 + 13 + 18
Environmental phobias	4 + 6 + 10 + 12
Blood-injection-injury phobias	11 + 15 + 19
Situational phobias/agoraphobia	5 + 7 + 16 + 17 + 21 + 22
Other phobias	2 + 8 + 14 + 20

### Summary of the YAM-5 Original Versions and the New Possible Combinations Available to Potential Users

**Table:**

a. YAM-5-I: original version measuring Major anxiety disorders (28 items)
b. YAM-5-II: original version measuring Specific phobias and Agoraphobia (22 items)
c. YAM-5-I+: which adds the Agoraphobia subscale to the YAM-5-I (34 items)
d. YAM-5-I-: excluding items of the Selective mutism subscale (24 items)
e. YAM-5-I-/+ : excluding items of the Selective mutism subscale but includes the Agoraphobia subscale (30 items)

To apply the YAM-5 (child and/or parent report version) and obtain the measure forms, either the original version or including specific combinations, please contact Prof. Peter Muris (Maastricht University): peter.muris@maastrichtuniversity.nl.
